# Contralateral bone conducted sound wave propagation on the skull bones in fresh frozen cadaver

**DOI:** 10.1038/s41598-023-32307-y

**Published:** 2023-05-09

**Authors:** Jihyeon Lee, Wan-Ho Cho, Tae Hoon Kong, Sung-Soo Jung, Woojae Han, Sihun Park, Young Joon Seo

**Affiliations:** 1 Department of Otorhinolaryngology-Head and Neck, Yonsei University Wonju College of Medicine, 20, Ilsan-ro, Wonju, Gangwon-do 26426 Republic of Korea; 2 Research Institute of Hearing Enhancement, Yonsei University Wonju College of Medicine, Wonju, South Korea; 3 Division of Physical Metrology, Korea Research Institute of Standard and Science, Daejeon, Republic of Korea; 4 Division of Speech Pathology and Audiology, Research Institute of Audiology and Speech Pathology, College of Natural Sciences, Hallym University, Chuncheon, Republic of Korea; 5 Department of Speech Pathology and Audiology, Graduate School, Hallym University, Chuncheon, Republic of Korea

**Keywords:** Bone, Biological physics, Medical research

## Abstract

The study aimed to investigate the efficient pathway for BC sound transmission by measuring vibrations on the opposite side of the skull bone, referred to as the mastoid position. The realistic contralateral transmission pathway of bone conduction (BC) vibrations is investigated through each osseous structure in the midlines of the fresh-frozen whole head. BC stimulation is applied to the mastoid using a bone vibrator, and acceleration responses are observed on the contralateral mastoid bone and seven midline points of skull bones using triaxial accelerometers. The study finds that the range showing the highest contralateral transmission efficiency of bone vibration is the intermediate frequency range with contralateral direction. Within this range, a significant amplitude of acceleration response is measured at the face-side points and the back and upper parts of the head. The thesis suggests that signal transmission from the specific midline to the mastoid can be more efficient than the conventional configuration of BC from the mastoid to the mastoid.

## Introduction

Understanding the complex mechanisms of bone conduction is crucial in improving the efficacy of bone-anchored hearing aids (BAHAs) for patients with unilateral hearing loss. BAHAs transmit sound to the contralateral ear, which helps overcome the head shadow effect and improves speech understanding^1^. Bone conduction mechanisms are complex and influenced by several factors, including signal frequencies. The suture of the skull can cause deformation of vibration patterns^2^, while bone thickness and density can impact sound propagation direction and vibration amplitude^3^. Ossicles play a role in bone conduction through piston or hinge movement, depending on the sound level^4^. Moreover, soft tissue in the skull also plays a significant role in bone conduction^5–8^. To optimize the effectiveness of BAHAs, understanding how sound reaches the contralateral ear is essential.

The contralateral bone conduction (BC) occurs through four different sound wave propagation mechanisms: (1) tangential and (2) normal to the skull bone surface, (3) rigid-body motion, and (4) direct propagation through the cerebrospinal fluid and brain tissue^9^. The dominant mechanism for contralateral bone conduction (BC) occurs through the skull bone when sound vibrations travel to the opposite side^10,11^. Stimulation of the mastoid initiates a vibration journey that can take various paths through the thin bony shell of the skull vault, thick skull base, or interior of the skull to reach the opposite ear^12^. The nature of cranial vibration modes varies according to the frequency range^13^. At around 300 Hz or below, the skull moves in a rigid body motion. For frequencies between 300 and 1000 Hz, the dynamics of the skull are a mass-spring system. At 1000–2000 Hz, there is a transition from a mass-spring system to a wave transmission. Above 2 kHz, the primary longitudinal wave transmission is primarily in the skull base and involves a mixed mode with a bending wave motion.

The aim of this study was to investigate the dominant pathway of contralateral sound transmission, including the anterior of the skull, using a freshly frozen cadaver to collect data under more realistic conditions.

## Material and methods

### Specimens

The investigation was conducted using the head of a fresh human cadaver cut from the body at the neck without any history of head trauma (male aged 54). The cadaver was not exsanguinated but was washed with an antiseptic soap before frozen at − 20 °C within a week of procurement. Thawing of the cadaver was done at room temperature for approximately three days before the study.

Measurements were made by placing a wooden support on the neck. When measuring the anterior part, it was measured on the back of the head, and when measuring the occipital part, it was measured with the forehead in contact with the surface (bed) made of soft cloth.

The study was conducted in accordance with Korean law as of the experiment date (July 24, 2019)^14^, the cadaver study did not need to obtain IRB approval. Additionally, both the donor and his family members had previously given their consent for the post-mortem use of the body for scientific research, in compliance with the human body donation system of Yonsei University College of Medicine.

### Measurement setup and procedure

Skin flaps containing muscle and periosteum of the skull midline points and left mastoid were made an incision to measure the vibrations directly from the bone. The seven points are as follows: (1) the chin point of the mandibular bone, (2) the nasion point of the nasal bone, (3) the forehead point of the frontal bone, (4) the upper-forehead point near the frontal-parietal bone suture, (5) upper and upper-forehead point of the parietal bone, (6) top point near the parietal-occipital bone suture, and (7) occipital point of the occipital bone. The detailed description of measurement position is shown in Supplemental Fig. 1.

Five triaxial accelerometers were used during this experiment (No. 1 to 4, Brüel & Kjær Type 4524B; No. 5, Brüel & Kjær Type 4535B). The 4525B is a 10 mm cube weighing 4.4 g and the 4535B is a 12 mm cube weighing 6 g. To minimize any structural changes to the skull, all accelerometers were attached to the measurement sites using a liquid superglue without the need for drilling or inserting needles. The bone vibrator was secured to the right mastoid using a steel headband with a static force of 5.5 N. Triaxial accelerometer No.1 was attached on top of the bone vibrator, while No. 5 was attached to the left mastoid. Furthermore, three triaxial accelerometers, No. 2, 3, and 4, were attached side by side on the point of the skull’s midline and moved to 7 different locations, ranging from the chin to the occipital, to observe the complex modal behavior and dominant pathway at the midline. In other words, while No.1 and No.5 were kept fixed, No. 2, 3, and 4 were moved in one set from one location to the next after the measurement was completed.

The measurement system employed two different coordinate systems: one for the mastoid positions and the other for the accelerometer positions at the midline. For the mastoid positions, the triaxial accelerometers (No. 1 and 5) were used to measure vibrations in three orthogonal axes—X-, Y-, and Z-axes. When triaxial accelerometers move along the midline (No. 2–4), the curvature of the head causes a misalignment between the accelerometer coordinates and the position of the vibrator or mastoid. To resolve this issue, the X-, Y-, and Z-axes of the triaxial accelerometers at the midline were redefined based on the sound propagation direction, using the elevation, azimuth, and normal directions. The elevation direction represents the direction of the top of the head, while the azimuth direction represents the contralateral direction from the right to the left ear. The normal direction is the line perpendicular to the tangent plane of the elevation, and the azimuth direction is the outer-right plane. The elevation and azimuth directions are longitudinal components propagating along the tangential plane, and the normal direction is the bending wave component.

The functional generator module of the DAQ (Brüel & Kjær LAN-XI Type 3160) produced excitation signals and was amplified by an audio amplifier (Alesis RA-100) used to supply the bone vibrations (B71). Two excitation signals were used: white noise excitation signals with a 12.8 kHz bandwidth with 6.4 kHz center frequency and continuous pure tone. The pure-tone excitation used frequencies of 250 Hz, 500 Hz, 1 kHz, 2 kHz, 4 kHz, 6 kHz, and 8 kHz.

The signal of the triaxial accelerometer was measured at a 12.8 kHz bandwidth (32.76 kHz sampling rate) and 8 Hz frequency resolution using two multichannel DAQ modules with 12 channels (Brüel & Kjær LAN-XI Type 3153). The acquisition time was 8.4 s. The measurements per point were repeated three times, and their means were used for analysis.

### Measured data

To compare the relative acceleration amplitude of vibrations measured at seven points along the midline of the skull, both pure-tone and random signals were used, and the auto-spectrum and frequency response were observed. The frequency response function (FRF) was obtained by dividing the cross-spectrum of the two signals by the auto-spectrum of the reference signal. This can be expressed as:

1 $${H}_{mn} \left(f\right)=\frac{{S}_{{A}_{n},{A}_{m}}\left(f\right)}{{S}_{{A}_{n},{A}_{n}}\left(f\right)},$$

Here S(f) denotes the power spectrum, and the subscripts An and Am represent the acceleration measured using the n-th and m-th accelerometers, respectively. To analyze the distribution of acceleration amplitude at each axis, the accelerometer at the exciter position (No. 1) was used as a reference point.

## Results

### Response at mastoid

**Figure 1:**
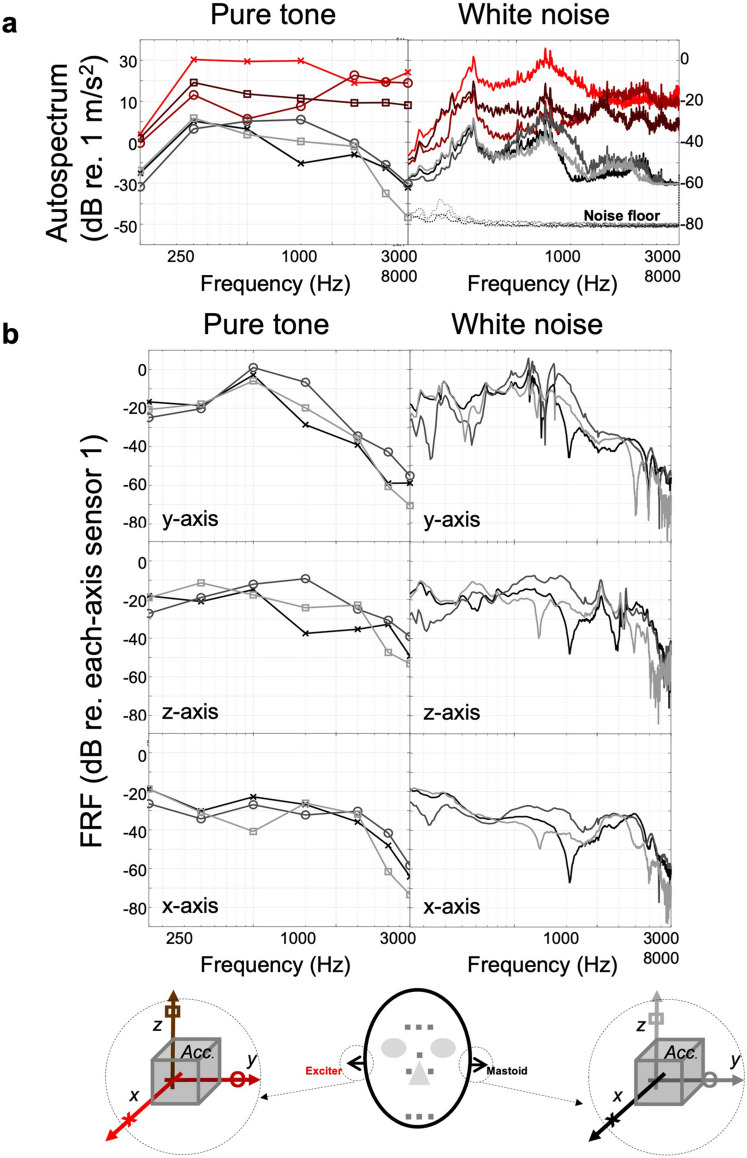
Measured vibration of cadaver head excited by the bone vibrator at mastoid position (The color and symbol of the curve correspond to the color and symbol of the direction of the arrow in the figure below): (**a**) autospectrum of the vibration of cadaver head excited by the bone vibrator at mastoid position, (**b**) frequency response (FR) of the cadaver head at the opposite mastoid positions excited by the bone vibrator at mastoid position.

Figure 1a shows an auto-spectrum at each mastoid position indicating the acceleration amplitude induced through a pure-tone excitation and white noise excitation (Fig. 1). The pure-tone excitation included frequencies of 250 Hz, 500 Hz, 1 kHz, 2 kHz, 4 kHz, 6 kHz, and 8 kHz, with the corresponding frequency components shown as connected curves. The y-direction was the major excitation axis due to the bone vibrator's structure, but the other axes' components had larger magnitudes than the y-axis components because those axes had no constrained condition in each direction. In the white noise response, two dominant peaks at 600 Hz and 1.2 kHz are observed, which can be induced by the frequency response of the bone vibrator.

To remove the transducer's characteristics and observe the relationship between each excitation and measurement position, the frequency responses referring to each axis of the excitation point were estimated and are shown in Fig. 1b (Fig. 1). The results showed that the excitation component of the y-direction was transferred more efficiently, especially at approximately 0.7–2 kHz than the other axis components. At the receiving mastoid position, the y-direction components were also relatively dominant in comparison with the other directions, particularly at approximately 2 kHz.

### Response at the midline

**Figure 2:**
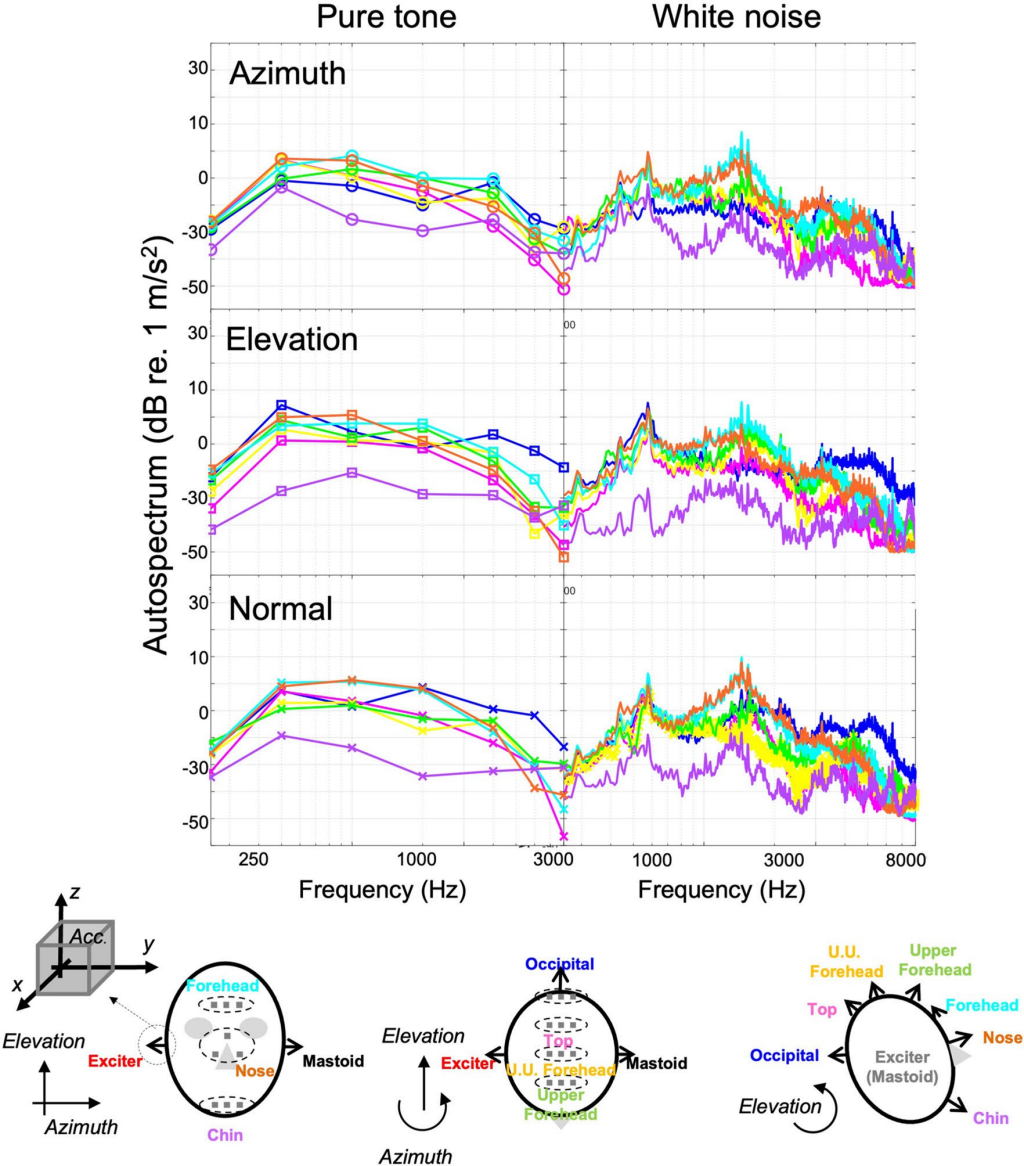
Autospectrum of the vibration of cadaver head excited by the bone vibrator on the midline. The averaged values of three accelerometers are shown for each direction.

**Figure 3:**
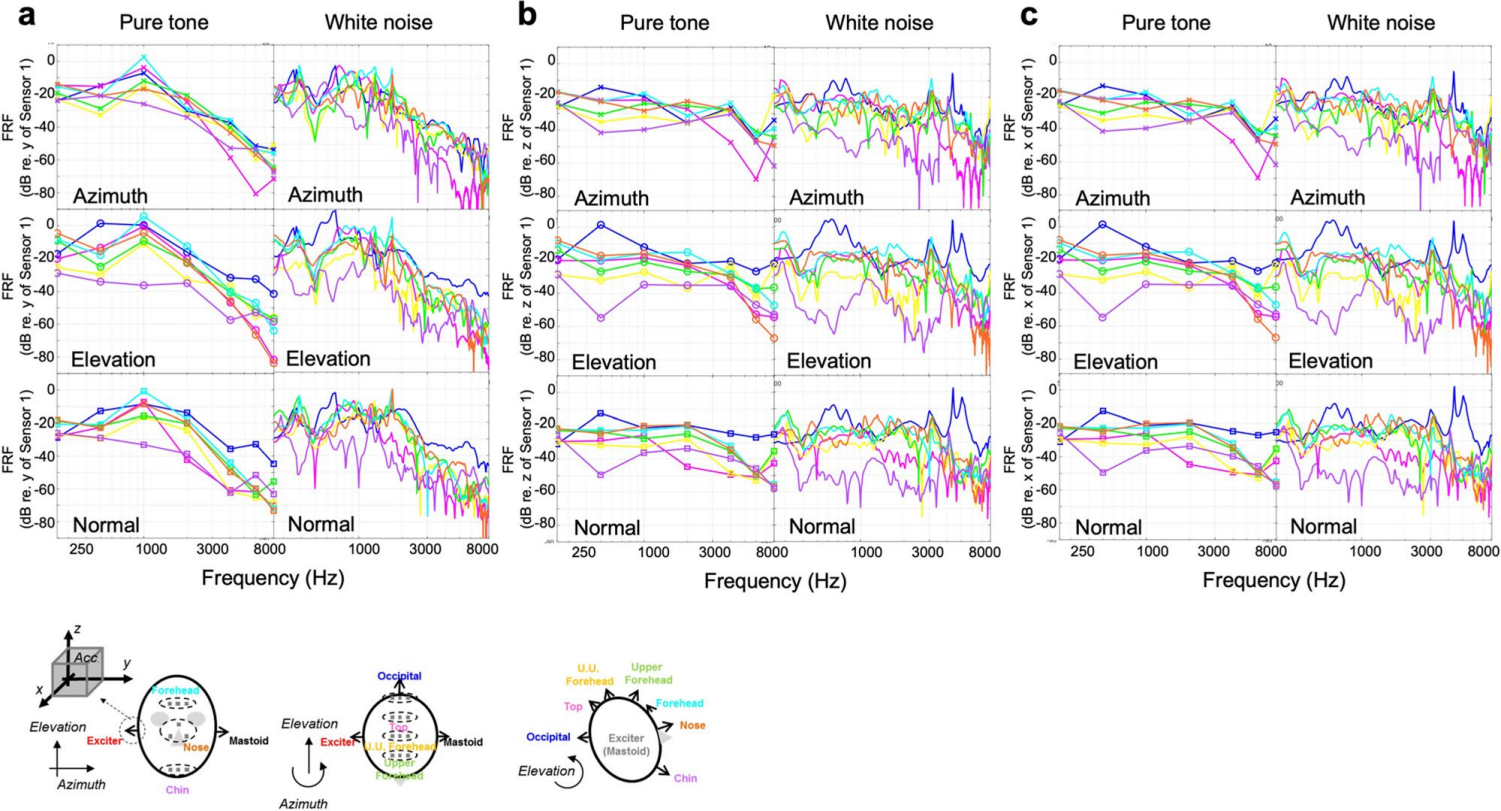
Frequency response (FR) of the cadaver head at the midline positions excited by the bone vibrator at mastoid position, referred to: (**a**) y-direction at excitation position, (**b**) z-direction at excitation position, (**c**) x-direction at excitation position. The averaged values of three accelerometers are shown for each direction.

A significant amount of the acceleration amplitude was measured at seven points on the skull midline (Figs. 2, 3). However, the acceleration amplitude at the chin was slightly lower than that of the other points overall. Excluding the chin, the acceleration amplitude of the anterior section of the skull, upper-forehead, and nose showed similar patterns, such as the temporal (contralateral mastoid), parietal (top, U.U. forehead), and occipital bone. Moreover, the y-direction components of the excitation point were observed to be efficiently transferred compared to the other directions, similar to the case between mastoid positions.

The skull thickness was measured by sagittal cutting the skull along the midline to investigate its effect on the measurements and is shown in Supplemental Fig. 2. The results showed that the chin bone was the thickest (7.6 ± 0.4 mm), while the nasal bone was the thinnest (2.3 ± 0.3 mm), and the other bones were similar in thickness (4.3–6.7 mm). Along, the azimuth axis, which had the greatest vibration efficiency on the opposite side, the acceleration amplitude transmitted past the chin was the lowest (Fig. 3).

### Phase differences at the midline of the skull bone

**Figure 4:**
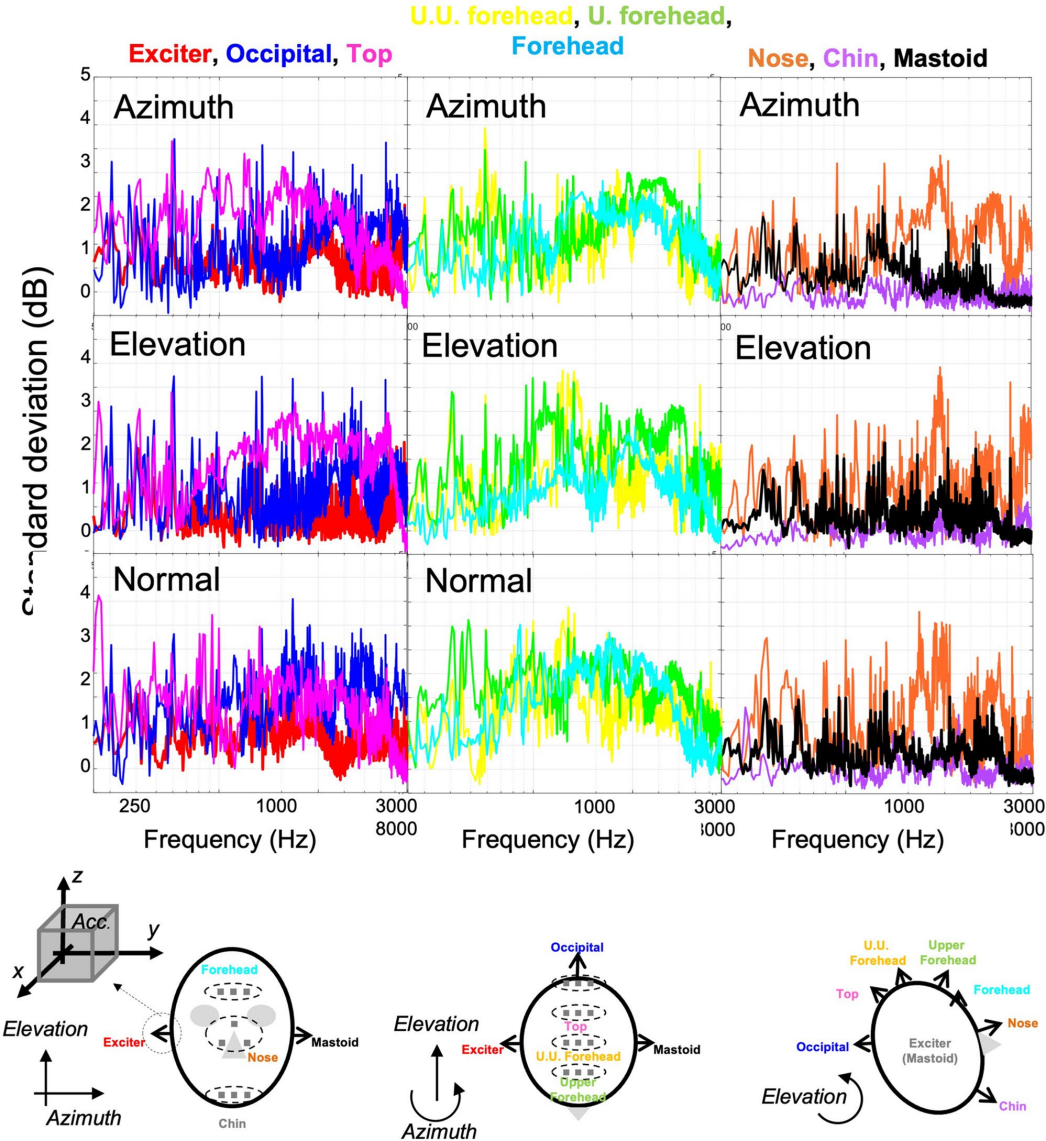
Standard deviation of the measured autospectrum of the vibration of cadaver head excited by the bone vibrator at mastoid position.

**Figure 5:**
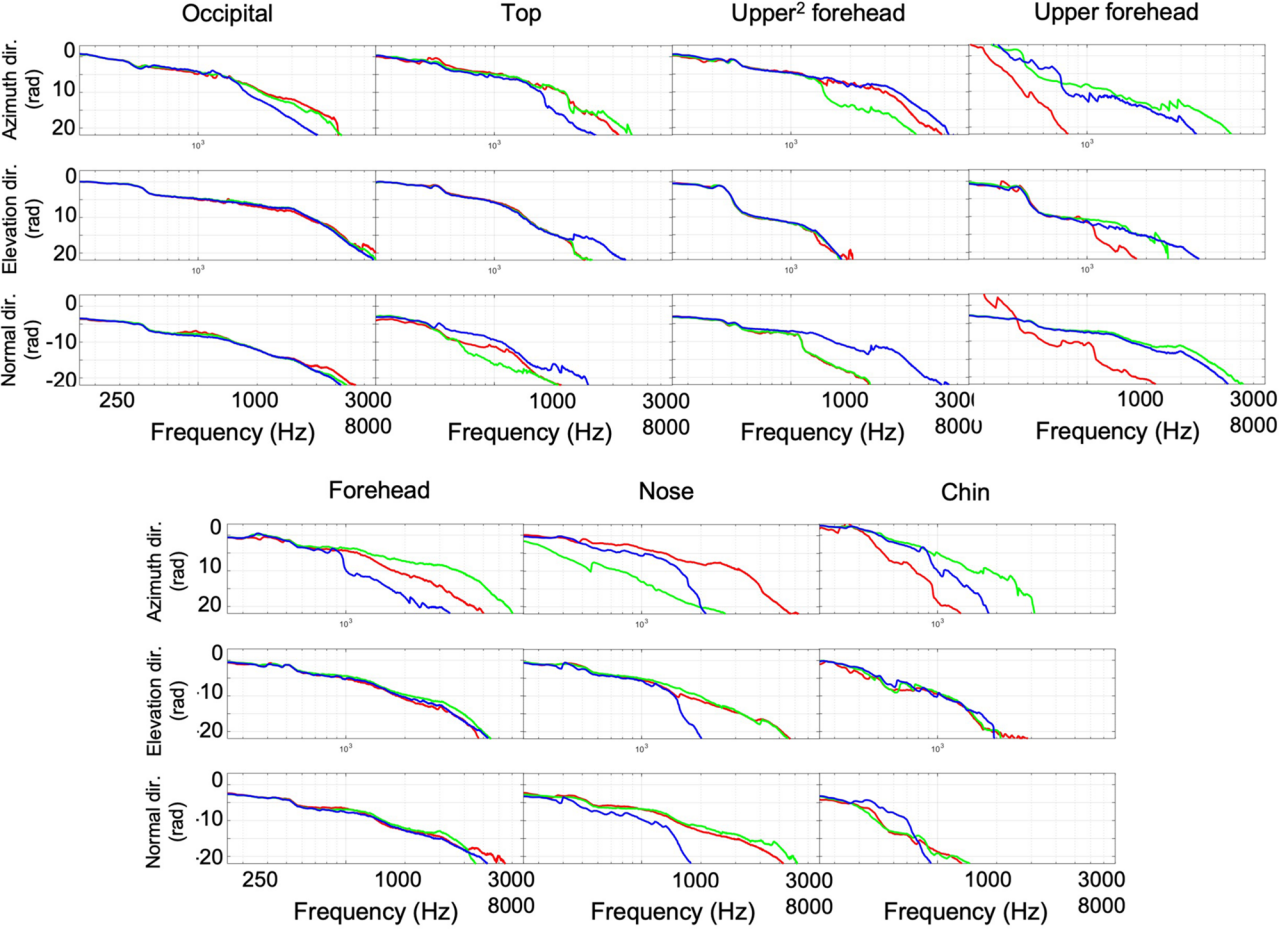
Comparison of the phase responses of cadaver head, referred to function generator output (Red, Acc. No. 2; Green, Acc. No. 3; Blue, Acc. No. 4).

The important characteristic observed in the response function is the phase difference between the seven points on the skull midline, which are referenced to the function generator output. As shown in Fig. 5, the phase difference between the three triaxial accelerometers (No. 2–4) attached to the skull midline was insignificant at or below 1 kHz (Fig. 4). This means that vibrations at low frequencies are transmitted as rigid body motion. At frequencies of above 1 kHz, the phase difference between the accelerometers became significant, indicating the revelation of higher-order vibration mode. This finding is consistent with previous studies that reported the first resonance frequency of the skull at approximately 1 kHz ^15^.

Because the occipital bone is a thick plate with a relatively simple structure, the phase difference on this position was higher than that of the other positions. On the other hand, the anterior side, such as the upper head, nose, and chin, showed increasing phase delay and phase difference between nearby measuring locations. This finding is attributed to the relatively complex bone structure in these points.

### Repeatability of signal and distortion

Overall, the signal to noise ratio was confirmed to be larger than 20 dB. To assess the repeatability of the measurement, the standard deviation of three measured autospectrum was observed, as shown in Fig. 5. In most of frequency range, the standard deviations were less than 3.5 dB.

**Figure 6:**
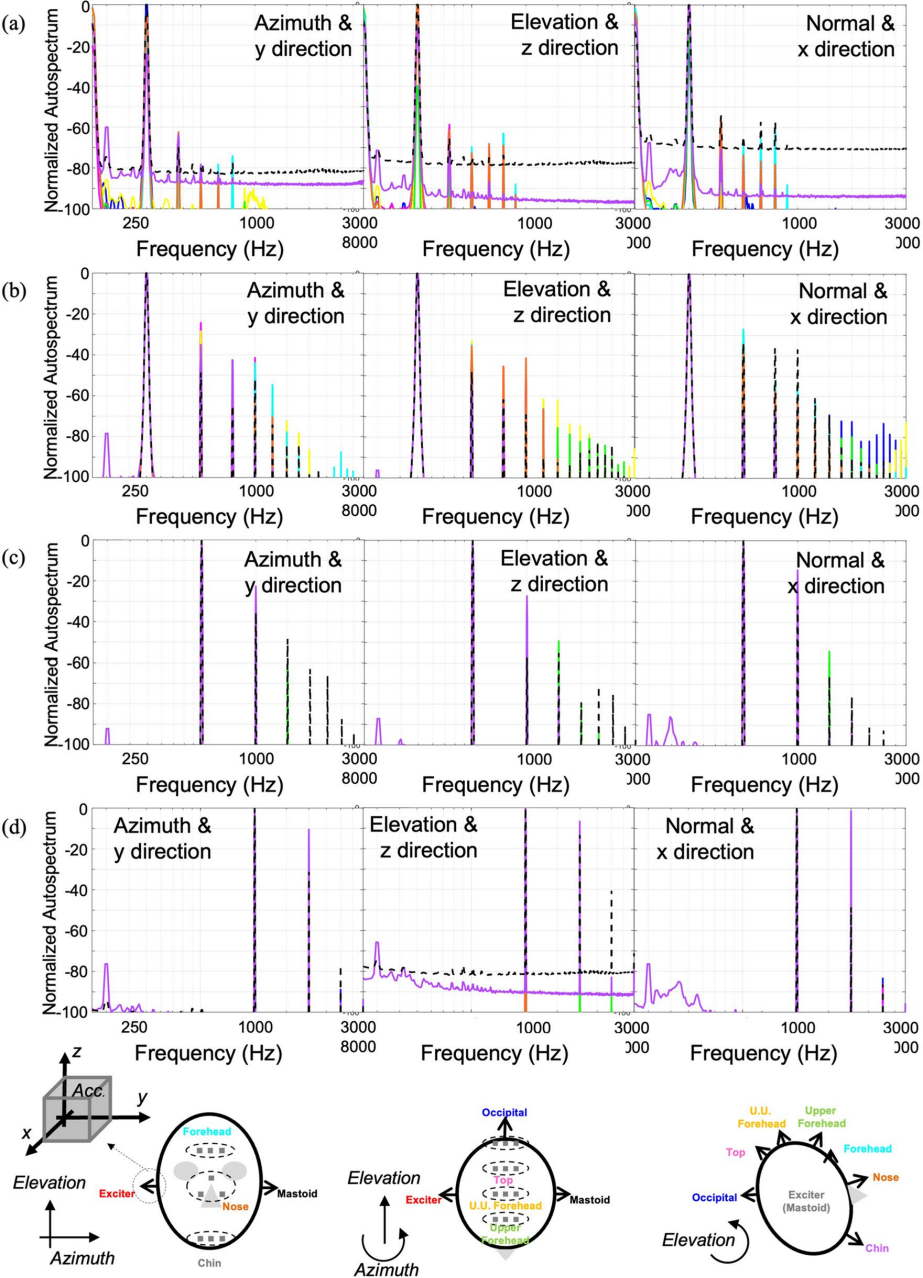
Normalized autospectrum of the vibration of cadaver head excited by the bone vibrator at mastoid position with pure tone signal: (**a**) 250 Hz, (**b**) 500 Hz, (**c**) 1 kHz, (**d**) 2 kHz.

Basically, the tendencies of pure tone excitation and white noise excitation are similar; however, they are not exactly the same because of harmonic distortion. Figure 6 shows a comparison of normalized autospectra induced by pure tone excitation for the entire frequency range. In the case of 250 Hz excitation, significantly large distortions were observed at the second harmonic, which is similar to or higher than the fundamental component. In a previous study^10^, it was reported that the nonlinearity of the head system is insignificant. The major cause of this high distortion could originate from the excessive amplitude of input having a large displacement owing to the same level of input was applied from the function generator. For the other higher frequency range, most of cases show that the level of second harmonic is lower than 20 dB, except for the response measured at the chin. This effect is much lower in the case of a random signal because the same level of r,m.s. input was supplied for wide band signal.

**Figure 7:**
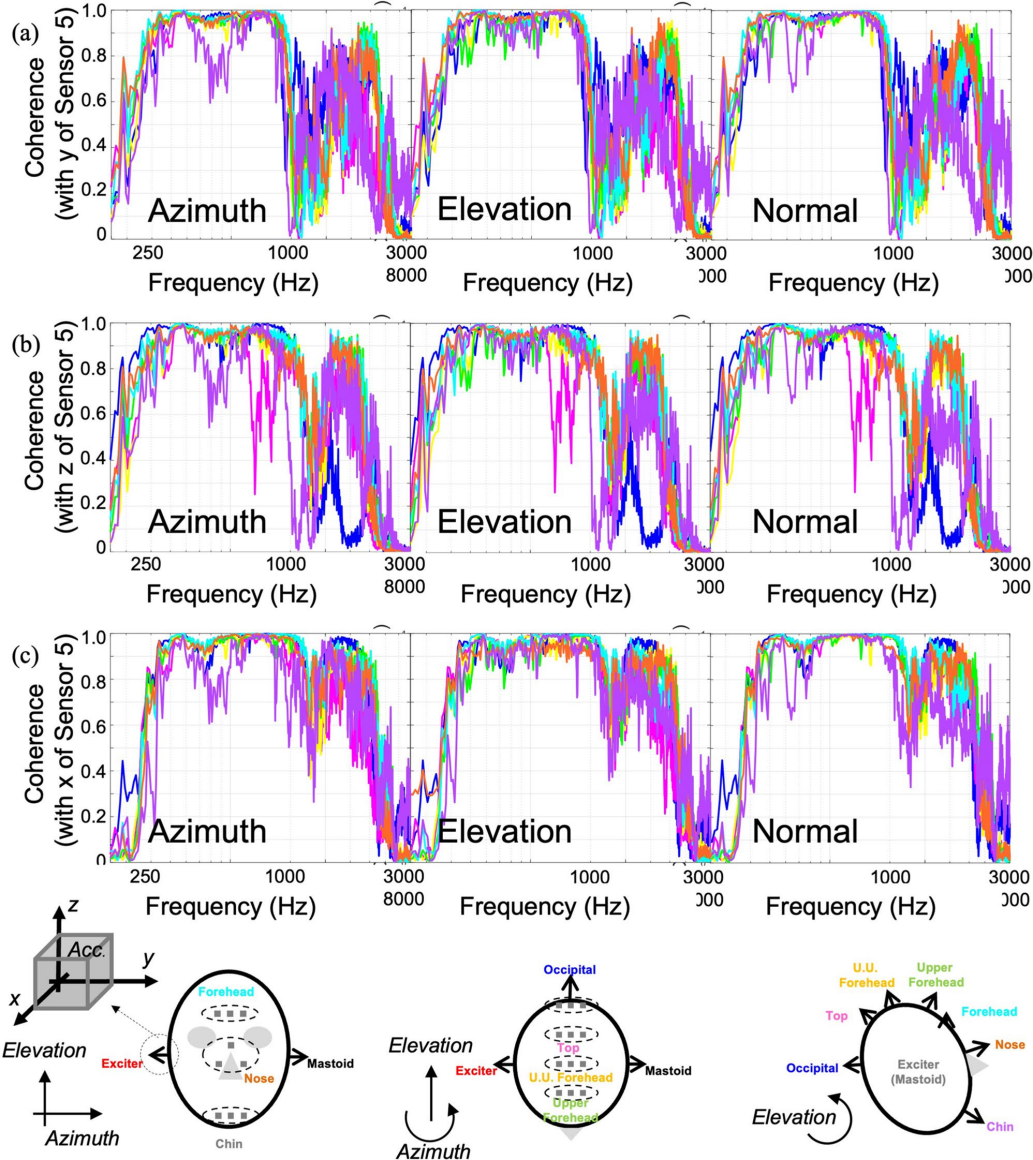
Coherence between each accelerometer output at the midlines of cadaver head excited by the bone vibrator at mastoid position and: (**a**) y-direction at mastoid position (Sensor 5), (**b**) z-direction at mastoid position (Sensor 5), (**c**) x-direction at mastoid position (Sensor 5).

The coherences between each accelerometer output on the midline position and mastoid position of excitation are also observed as shown in Fig. 7 and the coherence higher than 0.9 is observed in the frequency range of 0.5–2 kHz in overall. The chin and top positions shows relatively lower coherence than the other positions in several directions. This means that the efficient and coherent signal transmission is possible within the frequency range of 700 Hz to 2 kHz throughout not only backhead but also forehead side.

## Discussion

The BAHA is a hearing aid commonly used to treat patients with unilateral hearing loss by transmitting bone conduction sound to the contralateral ear. Our hypothesis was that the vibrations of various skull bones, including the facial bones, would vary based on the frequency, and transverse waves would appear differently in each bone. To test this, we measured the acceleration amplitude using a triaxial accelerometer at seven points along the skull midline of a human cadaver. The objective was to determine the vibration method and phase of bone conduction sound transmission.

The results of the measurements revealed a significant acceleration amplitude in the mid-frequency range at the facial points as well as at the back and upper regions of the head. This finding suggests that the contralateral bone conduction efficiency was high at the front of the face. These results, particularly at the frequencies related to higher mode, were in line with the transcranial attenuation of vibrations reported in a previous study^8^. The observation that sound propagation is efficiently transmitted not only to skull bones but also to facial bones provides valuable information for the application of bone conduction implants (BCIs) in patients with unilateral hearing loss. Moreover, the position that showed the highest level of response is an efficient location for transmitting or monitoring stimuli to the opposite mastoid.

It has been observed that the efficiency of bone conduction is reduced and the phase dispersion is greater at the chin, which may be due to the bone's structure and thickness. Unlike other parts of the skull, the jawbone is connected to the skull through a joint and is not firmly attached to the upper part of the skull. Additionally, it is thicker than other parts of the skull, and the mandibular bone, in particular, is relatively thick and has a temporomandibular joint that lowers the efficiency of bone conduction. According to a study by Eeg-Olofsson et al., the squamosal sutures between skull bones can affect sound transmission through bone conduction, resulting in an average damping effect of approximately 2 dB for frequencies above 2 kHz^8^. The efficiency of bone conduction depends on the thickness of the bone and its distance from the cochlea, and bone thickness can affect the frequency specifications of various pathways involved in hearing. Sohmer et al. reported that the threshold for bone conduction was lower when vibration was applied to the thin temporal bone than when applied to the thick forehead section of a dried cadaver^16^. Similarly, this experiment showed the lowest vibration efficiency in the thickest bone, which is the chin.

In the cranial vibration mode based on the phase difference at the skull midline, the rigid body motion was found to be dominant at frequencies of 300 Hz or less (less than 150–400 Hz), which is consistent with the previous study^13^. Additionally, above 2 kHz, wave transmissions dominated skull transitions, characterized by longitudinal wave propagation at approximately 400 m/s at the skull base and a mixture of vibration modes with 250–300 m/s, such as bending waves at the cranial vault.

Because the sample observed in this study was limited by single cadaver and it is not sufficient to draw quantitative and concrete conclusions. It is also required to measure the cochlear vibration to observe the effect on the actual delivered signal to auditory system. Nevertheless, the results presented the experimental evidence that the usage of midline position for observation and transferring the BC stimulation can be efficient for supporting the follow-up studies.

## Conclusion

The study aimed to investigate the most efficient pathway for BC sound transmission by measuring vibrations on the opposite side of the skull bone, referred to as the mastoid position. While previous studies have primarily considered the areas near the mastoid and occiput as excitation and monitoring points for bone conduction transmission, the study found that vibrations of the skull bones, including facial bones, vary in frequency and appear differently in each bone.

The study revealed that the intermediate frequency range with contralateral direction showed bone vibration's highest contralateral transmission efficiency. Additionally, the study found that stimulating the mastoid produced the most significant vibration, which was the most efficient position for transmitting or monitoring the vibration to the opposite mastoid. Furthermore, the study suggested that signal transmission from the specific midline to the mastoid can be more efficient than the conventional configuration of BC from the mastoid to the mastoid. There may be some difficulties in applying the current equipment as it is. However, it is meaningful to consider the development of a new methodology or device that can use these positions.

Overall, the study findings provide valuable insights into the efficient pathway for BC sound transmission and suggest that optimizing the excitation and monitoring positions can improve the efficacy of BC hearing aids.

## Supplementary Information

Supplementary Figure 1.

Supplementary Figure 2.

## Data Availability

The datasets generated during and/or analyzed during the current study are available from the corresponding author upon reasonable request.
