# Author Correction: Contralateral bone conducted sound wave propagation on the skull bones in fresh frozen cadaver

**DOI:** 10.1038/s41598-023-38126-5

**Published:** 2023-07-10

**Authors:** Jihyeon Lee, Wan‑Ho Cho, Tae Hoon Kong, Sung‑Soo Jung, Woojae Han, Sihun Park, Young Joon Seo

**Affiliations:** 1 Yonsei University Wonju College of Medicine, 20, Ilsan‑ro, Wonju, Gangwon‑do 26426 Republic of Korea; 2 Yonsei University Wonju College of Medicine, Wonju, South Korea; 3 Division of Physical Metrology, Korea Research Institute of Standard and Science, Daejeon, Republic of Korea; 4 Hallym University, Chuncheon, Republic of Korea; 5 Hallym University, Chuncheon, Republic of Korea

Correction to: *Scientific Reports* https://doi.org/10.1038/s41598-023-32307-y, published online 09 May 2023

The Funding section in the original version of this Article was incomplete.

“This study received funding from the Korean Fund for Regenerative Medicine (KFRM), a Grant funded by the Korean government (including the Ministry of Science and ICT and the Ministry of Health & Welfare) (21A0101L0). Additionally, partial support was provided by a Grant from the Korea Research Institute of Standards and Science (KRISS-2022-GP2022-0002-11).”

now reads:

“This study received funding from the Korean Fund for Regenerative Medicine (21C0721L1) funded by Ministry of Science and ICT and Ministry of Health and Welfare, and from the Technology Innovation Program (20016225, Development and Dissemination on National Standard Reference Data) funded by the Ministry of Trade, Industry & Energy(MOTIE, Korea). Additionally, partial support was provided by a Grant from the Korea Research Institute of Standards and Science (KRISS-2022-GP2022-0002-11)”.

The original Article has been corrected.

