# Supplementary figures and images for: Contralateral bone conducted sound wave propagation on the skull bones in fresh frozen cadaver
Source: Sci Rep. 2023 May 9;13:7479. doi: 10.1038/s41598-023-32307-y (PMC10169848; doi:10.1038/s41598-023-32307-y)

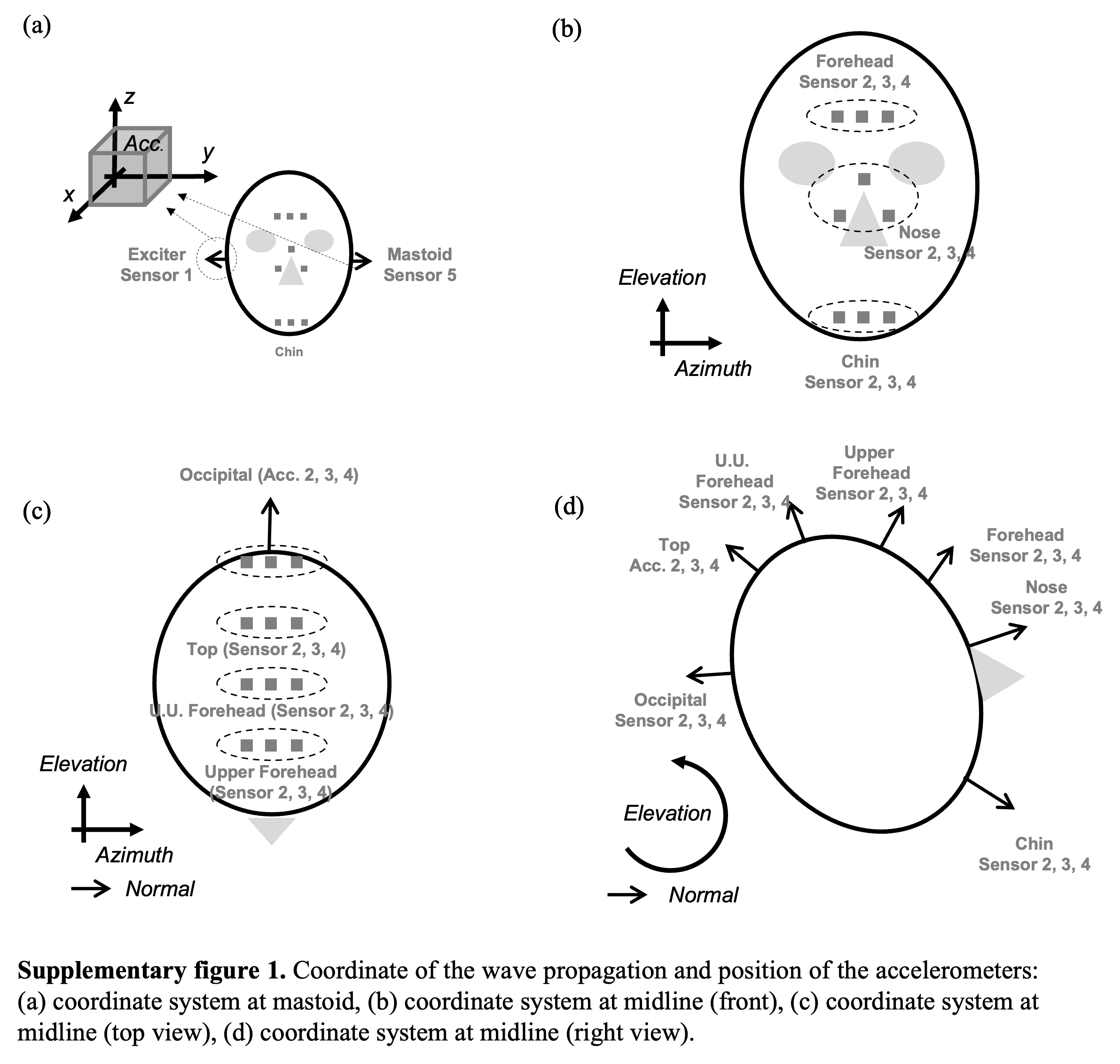

Supplement: Supplementary file 1 — Supplementary Figure 1. [file 41598_2023_32307_MOESM1_ESM.png]

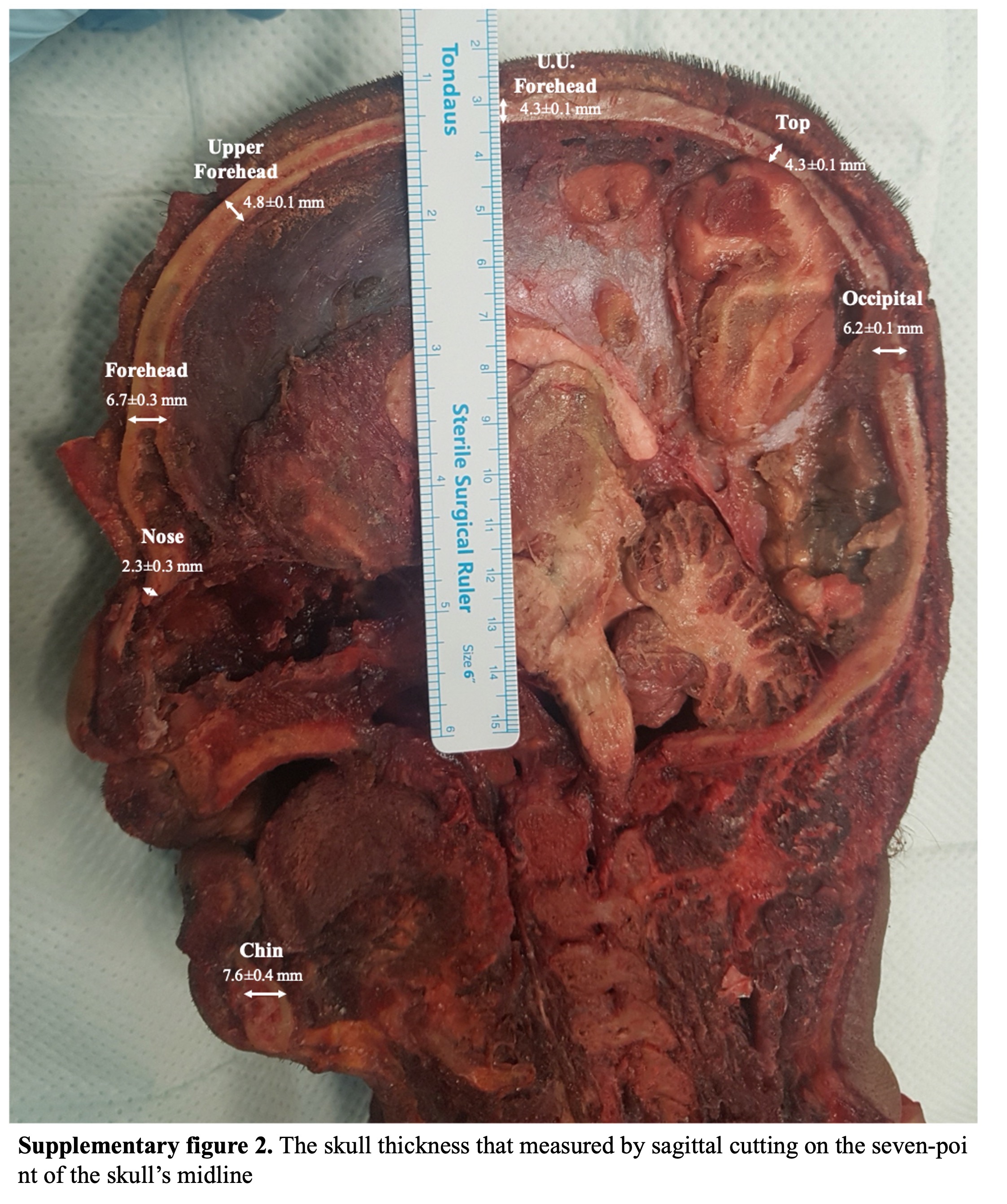

Supplement: Supplementary file 2 — Supplementary Figure 2. [file 41598_2023_32307_MOESM2_ESM.jpg]
